# Understanding the relevance of sample size calculation

**DOI:** 10.4103/0301-4738.71673

**Published:** 2010

**Authors:** Barun Kumar Nayak

**Affiliations:** P. D. Hinduja National Hospital and Medical Research Center, Veer Savarkar Marg, Mahim, Mumbai - 400 016, India. E-mail: editor@ijo.in

Research is done to find a solution to a particular medical problem (formulated as a research question which in turn is) based on statistics. In an ideal situation, the entire population should be studied but this is almost impossible. Other than census, which is conducted on each and every person of the population, all other studies are performed on limited subjects drawn from the concerned population known as “sample population”. The data obtained is analyzed and conclusions are drawn which are extrapolated to the population under study. The purpose of this editorial is to highlight the need and importance of sample size calculation which should be performed before starting any study.

The importance of sample size calculation cannot be overemphasized. A research can be conducted for various objectives. It may be done to establish a difference between two treatment regimens in terms of predefined parameters like beneficial effects, side effects, and risk factors of these regimens. It may also be carried out to prove similarity between groups. Sometimes, the purpose may be to achieve certain estimation in the population, such as the prevalence of a disease. Whatever be the aim, one can draw a precise and accurate conclusion only with an appropriate sample size. A smaller sample will give a result which may not be sufficiently powered to detect a difference between the groups and the study may turn out to be falsely negative leading to a type II error. A study on a small sample is quite tempting for obvious reasons, but it is a waste of time and money as the result will be invariably inconclusive. Very often, a small sample size is decided arbitrarily based on the researchers’ convenience, available time, and resources, resulting in a null trial due to insufficient number of subjects studied. Moher *et al*,[1] highlighted the magnitude of underpowered studies resulting in null trials in literature. In a study, they found that out of 102 null trials, only 36% had 80% power to detect a relative difference of 50% between groups. Only for a rare disease or indication is an underpowered study justified, due to logistics as the data from such a study is helpful in meta-analysis.

A very large sample size is also not recommended as it has its own consequences. First, it is a waste of the limited available resources in terms of time and money when an answer can be accurately found from a smaller sample. Secondly, recruiting more subjects than required can also be termed as unethical as the patients participate in a study with faith and an altruistic motive which should not be misutilized. Thirdly, in randomized controlled trials more people will be denied a better regimen and will get a placebo or an inferior treatment with its associated side effect or toxicity due to the inherent design of the study. These valid reasons are enough to justify proper sample size estimation before the start of any study.

Though sample size calculation may vary based upon the type of study design, the basic concept remains the same. The three main factors which must be considered are α-error, β-error and clinically significant difference or the effect size. Type I error or α-error is failure to accept the null hypothesis when it is actually true. Usually it is set at 5%. The sample size has to be increased if this value has to be lowered. Type II error or β-error is failure to reject the null hypothesis when it is not true. By convention, it can be set at 20%, 10% or 5%. Power of the study is equal to 1-type II error; hence any study should be at least 80% powered. The sample size increases when the power of study is increased from 80% to 90% or 95%. The third factor is the effect size. A small clinically significant difference is difficult to identify and needs a larger sample size as compared to a study with a larger clinically significant difference. The other factors which need to be considered are standard deviation for quantitative measurements, margin of error and attrition rate. These values are either known from literature or can be decided by a pilot study or by reasonable guess work. The number that we get after these calculations is not the exact figure but an approximate guide for the sample size. Sometimes, the sample size thus calculated has to be adjusted for feasibilities such as funds, duration of study and available subjects. But, there should not be major shift of sample size on these counts. The basis of sample size chosen in a particular study must be provided in the ‘materials and methods’ section of the paper for the benefit of its readers. Moher *et al*,[1] found in 1994 that only 32% null trials reported sample size calculations in published papers. The editors are now particular regarding the reporting of basis of sample size calculation in published papers. Any further discussion on the principles of sample size calculation is out of the scope of this editorial. However, the two articles of Malhotra *et al*,[2] and Gogate[3] in this issue of Indian Journal of Ophthalmology as well as some other key papers[1,4–9] will provide further insight in the understanding of sample size calculation.

Any major mistake in the sample size calculation will affect the power and value of a study. “Common sample size mistakes include not performing any calculations, making unrealistic assumptions, failing to account for potential losses during the study and failing to investigate sample size over a range of assumptions. Reasons for inadequately sized studies that do not achieve statistical significance include failing to perform sample size calculations, selecting sample size based on convenience, failing to secure sufficient funding for the project, and not using the available funding efficiently.”[6]

In conclusion, sample size calculation is a very important aspect of any study. It should be done at the time of planning a study, based on the type of the research question and study design. It is advisable to take the help of a statistician at this stage of the study as well. Authors must provide detailed information regarding the sample size calculation used when publishing their papers. Many null studies may be underpowered to detect the desired difference due to a smaller sample size. The underpowered studies should be interpreted cautiously and the ‘absence of evidence’ in these studies should not be taken as ‘evidence of absence’.
